# Diagnostic utility of oropharyngeal swabs as an alternative to lower respiratory tract samples for PCR-based syndromic testing in patients with community-acquired pneumonia

**DOI:** 10.1128/jcm.00505-23

**Published:** 2023-08-16

**Authors:** Sondre Serigstad, Siri T. Knoop, Dagfinn L. Markussen, Elling Ulvestad, Rune O. Bjørneklett, Marit H. Ebbesen, Øyvind Kommedal, Harleen M. S. Grewal

**Affiliations:** 1 Emergency Care Clinic, Haukeland University Hospital, Bergen, Norway; 2 Department of Clinical Medicine, University of Bergen, Bergen, Norway; 3 Department of Clinical Science, Bergen Integrated Diagnostic Stewardship Cluster, University of Bergen, Bergen, Norway; 4 Department of Microbiology, Haukeland University Hospital, Bergen, Norway; NorthShore University HealthSystem, Evanston, Illinois, USA

**Keywords:** community-acquired pneumonia, Biofire FilmArray Pneumonia panel, syndromic testing, sputum, oropharyngeal swab, upper respiratory tract sample, *Streptococcus pneumoniae*, *Haemophilus influenzae*, molecular diagnostics

## Abstract

Syndromic PCR-based analysis of lower respiratory tract (LRT) samples in patients with community-acquired pneumonia (CAP) improves the bacterial yield and time-to-results compared to culture-based methods. However, obtaining adequate sputum samples can be challenging and is frequently not prioritized in the emergency department (ED). In this study, we assess the concordance of microbiological detections between oropharyngeal- (OP) and LRT samples from patients presenting to the ED with CAP using a syndromic PCR-based respiratory panel [Biofire FilmArray Pneumonia *plus* (FAP *plus*)]. Paired OP- and high-quality LRT samples were collected from 103 patients with confirmed CAP, who had been included in a randomized controlled trial (NCT04660084) or a subsequent observational study at Haukeland University Hospital, and analyzed using the FAP *plus*. The LRT samples were obtained mainly by sputum induction (88%). Using the LRT samples as a reference standard, the positive percent agreement (PPA), negative percent agreement (NPA), and overall percent agreement for the most common bacterial pathogens in CAP, *Streptococcus pneumoniae* and *Haemophilus influenzae*, were 85%, 99% and 95%, and 86%, 98% and 93%, respectively. For *Moraxella catarrhalis,* the PPA was lower (74%), while the NPA was 100%. For bacteria that are less likely causes of uncomplicated CAP (e.g., *Staphylococcus aureus* and Enterobacterales) the results were more divergent. In conclusion, the FAP *plus* detects the most common CAP pathogens *S. pneumoniae* and *H. influenzae* from OP samples with high PPAs and excellent NPAs when compared with LRT samples. For these pathogens, the PPAs for OP samples were higher than previous reports for nasopharyngeal samples. This suggests that analysis of OP samples with syndromic PCR panels could represent an alternative approach for rapid microbiological testing in the ED, especially in patients where LRT samples are difficult to obtain. Divergent results for bacteria that are less likely to cause uncomplicated CAP do, however, emphasize the need for clinical evaluation of positive test results.

## INTRODUCTION

Community-acquired pneumonia (CAP) is one of the leading causes of hospital admissions and deaths in the world (1 - 3). Until recently, detection of typical respiratory bacterial pathogens relied mainly on sputum culture, which can be slow and insensitive. Studies using conventional microbiological diagnostic methods have found a plausible etiology in about 30%–50% of hospitalized pneumonia patients (4, 5).

The introduction of syndromic molecular assays with broad panels targeting common respiratory tract pathogens improves the microbiological yield and time to results when analyzing specimens from the lower respiratory tract (LRT) (6 - 10). Nevertheless, adequate sputum sampling is often difficult and time-consuming and thus frequently not prioritized in the emergency department (ED).

Three recent studies have evaluated the performance of syndromic PCR panels for CAP on samples from the upper respiratory tract (URT) (11 - 13). In all three studies, the results were compared against results obtained on LRT samples from the same patients. Two studies used TaqMan array card technology on combined oropharyngeal- (OP) and nasopharyngeal (NP) samples and reported inconsistent results (12, 13). The third study used the FAP *plus* on NP samples and demonstrated a high negative percent agreement (NPA). However, the positive percent agreement (PPA) was substantially lower, indicating an uncertain utility for withholding antibiotics in the case of negative results (11).

OP samples are easier and faster to obtain than both LRT- and NP samples. OP samples have demonstrated a higher sensitivity than NP samples for detection of atypical bacteria, but only a few studies with discordant results have examined the utility of OP samples for the detection of other CAP-related bacteria (14 - 19). To our knowledge, no study has compared detections from OP- with LRT samples using comprehensive PCR-based panels. We, therefore, aimed to examine the microbiological yield and concordance of detections by use of a syndromic multiplex respiratory panel in paired OP- and high-quality LRT samples from patients presenting to the ED with CAP.

## MATERIALS AND METHODS

### Patients and study design

We analyzed samples from prospectively enrolled CAP patients at Haukeland University Hospital, a tertiary care referral center in Bergen, Norway. The patients had been included in a randomized controlled trial (RCT) (CAPNOR, NCT04660084) or a following observational study on ED patients with suspected CAP, between October 1, 2020, and September 19, 2022. The criteria for eligibility were the same in both cohorts (20). In short, patients were considered for inclusion if they were ≥18 years, presenting to the ED with a suspicion of CAP and fulfilling at least two of the following criteria: new or worsening cough; new or worsening expectoration of sputum; new or worsening dyspnea; hemoptysis; pleuritic chest pain; radiological evidence of pneumonia; abnormalities on chest auscultation and/or percussion; fever (≥38.0°C). Exclusion criteria were cystic fibrosis, severe bronchiectasis, hospitalization within the last 14 days prior to admission, a palliative approach (defined as life expectancy below two weeks), or if the patient was not willing or able to provide an LRT sample. For the current investigation, all participants with a confirmed diagnosis of CAP were considered, provided that a paired OP- and high-quality LRT sample were available for analysis. The diagnostic criteria for CAP in the CAPNOR RCT have been described elsewhere (20, 21).

### Microbiological methods and sampling

Study nurses collected microbiological samples shortly after presentation to the ED. First, an OP sample was collected using an OP swab (Sigma VCM MW910PF) as part of routine hospital care (swabbing the OP back wall for approximately five seconds). Subsequently, an LRT sample was obtained from all patients (20, 21). Depending on clinical symptoms, vital signs, and medical history, either spontaneous sputum or sputum induced by either nebulized isotonic (0.9%) or hypertonic (5.8%) saline was collected. Patients with known obstructive lung disease and patients with hypoxemia or signs of airway obstruction upon physical examination were treated with a bronchodilator (salbutamol and/or ipratropium bromide) prior to sampling. If sputum induction was unsuccessful, we collected a sample by endotracheal aspiration. The LRT samples were evaluated both macroscopically upon sampling and were submitted for further analysis if visually mucoid/purulent. Microscopy was performed on LRT samples from all patients according to international guidelines (20, 22). A purulent looking portion was Gram stained, and samples with <10 squamous epithelial cells (SECs) or a ratio of leukocytes/SECs ≥10 and >5 microbes per field at a ×100 magnification were classified as high-quality. Only patients with high-quality LRT samples were included in this study.

Both LRT samples and the OP samples were analyzed with the Biofire FilmArray Pneumonia *plus* panel (FAP *plus*). All OP samples had been stored at −80°C prior to analysis, as had approximately half of the LRT samples that originated from the CAPNOR RCT. Because freezing is not listed as an approved storage method in the FAP *plus* manual, we checked the impact of freezing by performing repeated analysis on thawed LRT specimens from 22 consecutively enrolled CAP patients who initially had received immediate FAP *plus* testing on fresh samples (Table S2). Otherwise, the manufacturer’s instructions for sputum-like specimens were followed (23). For highly viscous samples, 500 µL 0.9% saline was added before running the FAP *plus*. For the OP samples, we used the Biofire sample swab to collect material from the Sigma VCM transport medium.

### Statistical analysis

Descriptive statistics for continuous variables are reported as medians with interquartile range (IQR). Fisher’s exact test was used for analyzing categorical data, by use of contingency tables. A two-tailed *P*-value ≤0.05 was considered statistically significant for all analyses. We calculated the PPA, the NPA, and the overall percent agreement (OPA, as well as the positive and negative predictive values (PPV and NPV) of the FAP *plus* in OP samples, using the FAP *plus* results of the LRT sample as a reference. The statistical analyses were performed using IBM SPSS Statistics (version 26.0; Armonk, NY, USA), GraphPad Prism (GraphPad Software, La Jolla, CA, USA), and the GraphPad QuickCalcs website: https://www.graphpad.com/quickcalcs/contingency1/ (last accessed March 10, 2023).

## RESULTS

### Patient characteristics and samples

**Fig 1:**
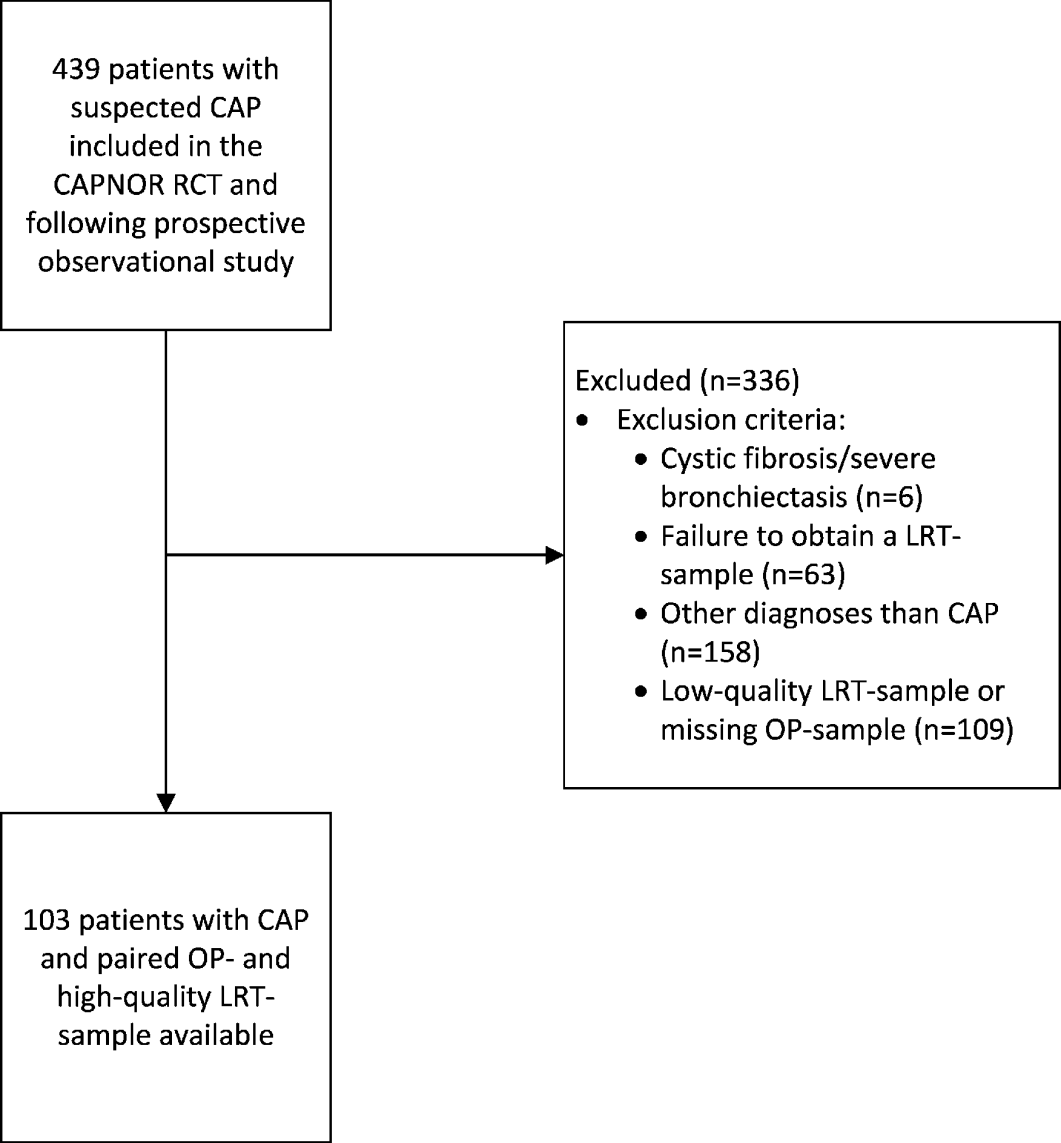
Study flowchart. *Abbreviations*: CAP, community-acquired pneumonia; LRT, lower respiratory tract; OP, oropharyngeal.

From a cohort of 439 ED patients with suspected CAP, we identified 103 patients with a retrospectively confirmed CAP and an available high-quality sputum sample with a paired OP sample (Fig. 1). The LRT samples were obtained mainly by sputum induction [88%, (91/103)], followed by spontaneous sputum [9% (9/103)] and endotracheal aspiration [3% (3/103)]. The median time between sampling of OP- and LRT samples in the ED was 65 (47–87) minutes. When presenting to the ED, 22% (23/103) of the patients had already started empirical oral antibiotics. Further patient characteristics are presented in Table 1.

**TABLE 1:** Characteristics of the study cohort^*a*^ ^*b*^

	CAP patients (*n* = 103)
Baseline characteristics	
Demography	
Age	70 (60–78)
Female	49 (48)
Male	54 (52)
Previous smoker	48 (47)
Current smoker	18 (18)
Comorbidity	
Hypertension	40 (39)
Heart failure	14 (14)
Diabetes mellitus	7 (7)
Asthma	15 (15)
COPD	40 (39)
COPD GOLD 1–2	7 (7)
COPD GOLD 3–4	22 (21)
COPD GOLD unknown	11 (11)
Vaccine status	
Influenza virus^*c*^	58 (56)
Pneumococcal^*d*^	41 (40)
Severity	
CURB-65	1 (1–2)
PSI score	83 (66–106)^*e*^
Clinical frailty score	3 (2–4)
CCI score	4 (2–5)
Highest WBC count	13.8 (10.5–19.0)
Highest CRP level	212 (138–293)

^*a*^ Data shown as count (%) or median (IQR).

^*b*^ *Abbreviations*: CAP, community-acquired pneumonia; COPD, chronic obstructive pulmonary disease; CURB-65, confusion, urea, respiratory rate, blood pressure, age ≥65 years; PSI, pneumonia severity index; CCI, Charlson Comorbidity Index; WBC, white blood cell; CRP, C-reactive protein; IQR, interquartile range.

^*c*^ Vaccinated for influenza virus within the preceding year.

^*d*^ Vaccinated within the last five years.

^*e*^ Missing for five CAP patients.

### Microbiological detections

**Fig 2:**
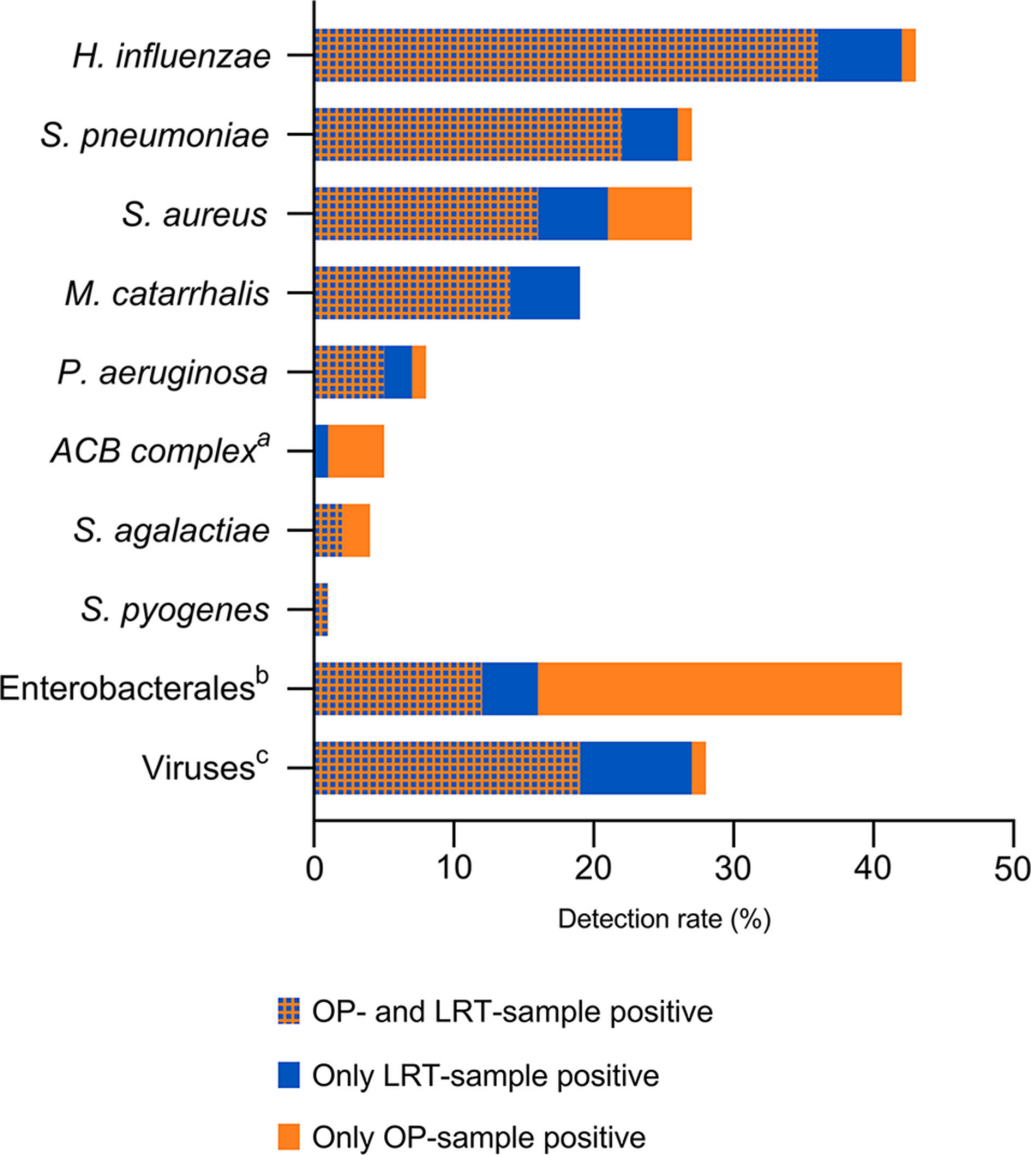
Detection rates by the Biofire FilmArray Pneumonia *plus* panel in paired OP and LRT samples from patients with CAP. Stratified by type of specimen. *^a^ Acinetobacter calcoaceticus-baumannii* complex. ^b^ *Enterobacter cloacae* complex, *Escherichia coli, Klebsiella aerogenes, Klebsiella pneumoniae, Klebsiella oxytoca, Serratia marcescens, Proteus* species. ^c^ Adenovirus, coronavirus (229E, OC43, HKU1, and NL63), parainfluenza virus, rhino-/enterovirus, RS virus, human metapneumovirus, influenza virus. *Abbreviations*: OP, oropharyngeal; LRT, lower respiratory tract; CAP, community-acquired pneumonia.

As illustrated in Fig. 2, *Haemophilus influenzae, Streptococcus pneumoniae,* and *Staphylococcus aureus* were the most frequently detected bacteria in both OP- and LRT samples. Rhino-/enterovirus was the most frequent viral detection, followed by respiratory syncytial (RS) virus and seasonal corona virus. A complete overview of results from all paired samples is provided in Table S1. The FAP *plus* panel detected one or more potential pathogen for 90% (93/103) of the patients. Lack of detections was not found to be associated with ongoing use of antibiotics [10% (8/80) vs 9% (2/23), difference of 1%, 95% CI −20% to 13%; *P* > 0.99].

Table 2 presents the concordance between detections in the OP samples and the LRT samples. When using the results from the LRT samples as the reference standard, the PPA, NPA, and OPA for *S. pneumoniae* and *H. influenzae*, the two most common pathogens in CAP, were 85%, 99%, and 95%, and 86%, 98%, and 93%, respectively. For *Moraxella catarrhalis* the PPA was lower (74%), while the NPA and the OPA remained high (100% and 95%, respectively). Detections made exclusively in the OP samples were primarily bacteria not normally associated with CAP (Enterobacterales and nonfermentative bacteria), as well as *S. aureus* (Table 2; Fig. 2).

**TABLE 2:** Performance and concordance between detections made by the Biofire FilmArray Pneumonia *plus* panel in paired OP- and LRT samples (*n* = 103)^*a*^ ^*b*^

Targets	OP and LRT positive	Only LRT positive	Only OP positive		Total negative	PPA	NPA	OPA	PPV	NPV
*H. influenzae*	36	6	1		60	86%	98%	93%	0.97	0.91
*S. pneumoniae*	22	4	1		76	85%	99%	95%	0.96	0.95
*S. aureus*	16	5	6		76	76%	93%	89%	0.73	0.94
*M. catarrhalis*	14	5	0		84	74%	100%	95%	1	0.94
*E. coli*	8	0	5		90	100%	95%	95%	0.62	1
*K. oxytoca*	1	0	10		92	100%	90%	90%	0.09	1
*P. aeruginosa*	5	2	1		95	71%	99%	97%	0.83	0.98
*S. marcescens*	0	1	6		96	0%	94%	93%	0	0.99
*E. cloacae complex*	1	2	2		98	33%	98%	96%	0.33	0.98
*ACB complex*	0	1	4		98	0%	96%	95%	0	0.99
*K. pneumoniae* group	1	1	3		98	50%	97%	96%	0.25	0.99
*S. agalactiae*	2	0	2		99	100%	98%	98%	0.50	1
*S. pyogenes*	1	0	0		102	100%	100%	100%	1	1
*Proteus* spp.	1	0	0		102	100%	100%	100%	1	1
*K. aerogenes*	0	0	0		103	N.A.	100%	100%	N.A.	1
Rhino-/enterovirus	7	4	0		92	64%	100%	96%	1	0.96
RS virus	5	1	0		97	83%	100%	99%	1	0.99
Coronavirus (229E, OC43, HKU1, and NL63)	4	1	1		97	80%	99%	98%	0.80	0.99
Parainfluenza virus	1	1	0		101	50%	100%	99%	1	0.99
Adenovirus	1	0	0		102	100%	100%	100%	1	1
Human metapneumovirus	1	0	0		102	100%	100%	100%	1	1
Influenza A virus	0	1	0		102	0%	100%	99%	N.A.	0.99

^*a*^ The detections from the LRT sample are used as a reference standard for calculating PPA, NPA, OPA, PPV and NPV.

^*b*^ *Abbreviations*: OP, oropharyngeal; LRT, lower respiratory tract; PPA, positive percent agreement; NPA, negative percent agreement; OPA, overall percent agreement; PPV, positive predictive value; NPV, negative predictive value; *ACB complex*, *Acinetobacter calcoaceticus-baumannii complex*; N.A., not applicable.

Table 3 displays a summary of the FAP *plus′* semi-quantitative results for cases with the same detected bacterium in the paired samples (LRT and OP). Most detections of *H. influenzae*, *S. pneumoniae* and *M. catarrhalis* had higher [53% (19/36); 64% (14/22); and 50% (7/14), respectively] or equal [36% (13/36); 32% (7/22); and 43% (6/44), respectively] semi-quantitative values in the LRT sample compared to the OP sample. Detections of Enterobacterales and *S. aureus*, but not *Pseudomonas aeruginosa*, tended in the opposite direction (Table 3).

**TABLE 3:** Overview of the FAP *plus′* quantitative results in patients where both the OP- and LRT samples detect the same microbe^*a*^

Targets	Quantitative values		
	Equal	Higher in LRT sample	Higher in OP sample
*H. influenzae*	13 (36%)	19 (53%)	4 (11%)
*S. pneumoniae*	7 (32%)	14 (64%)	1 (5%)
*S. aureus*	8 (50%)	2 (13%)	6 (38%)
*M. catarrhalis*	6 (43%)	7 (50%)	1 (7%)
*P. aeruginosa*	2 (40%)	3 (60%)	0 (0%)
*S. agalactiae*	1 (50%)	0 (0%)	1 (50%)
*S. pyogenes*	1 (100%)	0 (0%)	0 (0%)
*ACB complex*	0 (0%)	0 (0%)	0 (0%)
*Enterobacterales*	3 (25%)	0 (0%)	9 (75%)

^*a*^ *Abbreviations*: FAP *plus*, Biofire FilmArray Pneumonia *plus* panel; OP, oropharyngeal; LRT, lower respiratory tract; *ACB complex*, *Acinetobacter calcoaceticus-baumannii complex; Enterobacterales: Enterobacter cloacae complex, Escherichia coli, Klebsiella aerogenes, Klebsiella pneumoniae, Klebsiella oxytoca, Serratia marcescens, Proteus* species.

For viral detections, the OP samples demonstrated a high NPA, but a variable PPA when compared against the LRT samples (Table 2; Fig. 2).

## DISCUSSION

In this study, we evaluated the performance of a commercial rapid syndromic multiplex PCR panel on OP samples and high-quality LRT samples obtained from a well-characterized cohort of patients presenting to the ED with CAP. The PCR panel is validated for LRT samples, which can be difficult and time-consuming to obtain, especially in an ED setting. We demonstrated high NPAs and PPAs between OP- and high-quality LRT samples for the most common bacterial pathogens involved in CAP. To our knowledge, this study is the first to evaluate syndromic PCR testing on OP samples from CAP patients.

In previous CAP studies, an LRT sample has been obtained from only 30%–60% of the included patients, often after transfer from the ED (4, 16, 17, 24). Samples from the URT are much easier to collect and are frequently included in the initial diagnostic workup of CAP patients to test for viral and atypical bacterial pathogens. A few other studies have compared PCR-based testing for bacterial targets in URT samples versus LRT samples of adult patients with LRT infections. Important differences from our investigation include a smaller selection of targeted pathogens, use of NP- or combined NP/OP samples, and use of standard bacterial culture as the LRT reference (11 - 15 - 25 - 27 - 27). In line with our results, they all found a high NPA, whereas the PPA varied considerably. Two European studies that looked at the performance of real-time PCR for detection of *S. pneumoniae* in NP specimens found a PPA of 44 and 72%, respectively: considerably lower than our PPA of 85% for OP samples (26, 27). Three other studies have examined syndromic PCR-based testing of paired URT- and LRT samples (11 - 13). One study from the United States used TaqMan array card technology on combined NP/OP samples (12). Compared to our results, they found lower PPAs, ranging from 53% to 79% for *S. pneumoniae*, *H. influenzae*, and *M. catarrhalis*. Likewise, a study from Kenya that also used TaqMan array card technology on combined OP/NP samples found PPAs in the range of 43% to 80% for *S. pneumoniae*, *H. influenzae*, *M. catarrhalis,* and *S. aureus*, but their results are less readily comparable to ours due to a different epidemiological setting (13). Of note, the FAP *plus* was used in a recently published Swiss study that compared NP- and LRT samples in hospitalized CAP patients. The calculated PPAs for *S. pneumoniae*, *H. influenzae,* and *M. catarrhalis* in this study were substantially lower than ours (11). A delay of up to 24 h between the collection of URT- and LRT samples, as well as inclusion of samples up to 48 h after admission was accepted in the Swiss study. In addition to the likely impact of the time difference itself, this implicate that a significant proportion of their patients had started empiric antibiotics prior to sample collection. The Swiss study also excluded patients who had been hospitalized within the last three months (11). This might have imposed a selection bias, as their study cohort thereby most likely differ from a general CAP population with often relatively frequent hospitalizations. In comparison, we limited exclusion due to re-admissions to patients hospitalized during the last 14 days.

Pneumonia is a heterogeneous condition, with differences in microbiological etiology. The FAP *plus* is designed to be used in all pneumonia patients, including hospital- and ventilator-associated cases, explaining the high number of targets. *S. pneumoniae*, *H. influenzae,* and *M. catarrhalis* are recognized as the most frequent pathogens in a community-acquired setting, where particularly *M. catarrhalis* is associated with underlying chronic lung disease (28 - 30). We found that OP detections of *S. pneumoniae, H. influenzae,* and *M. catarrhalis* had excellent NPAs and PPVs when compared to a paired LRT sample. This means that a positive detection in an OP sample is likely to be reproduced in a paired LRT sample, justifying initiation of targeted antimicrobial treatment based on OP results for these microbes. In addition, the PPAs of *S. pneumoniae* and *H. influenzae* were high, with high NPV, indicating a potential to withhold antibiotics based on a negative OP sample in uncomplicated cases. *M. catarrhalis,* on the other hand, demonstrated a quite low PPA. A possible contributor to this result is colonization with *M. catarrhalis* in the LRT (28 - 30). Among the five patients where an LRT detection of *M. catarrhalis* was not reproduced in the OP sample, three had a predisposing lung disease. Importantly, *M. catarrhalis* was found in combination with the more plausible pathogen *S. pneumoniae* in three of these samples with concentrations just above the FAP *plus’* cut-off limit. For *S. aureus*, the PPA for an OP sample detection was also lower, as was the NPA. Compared to *S. pneumoniae* and *H. influenzae*, *S. aureus* is a less likely causative agent of uncomplicated CAP and a more likely colonizer of the URT (31 - 35). Indeed, *S. aureus* was often detected with higher semi-quantitative values in the OP samples compared with the LRT samples, further indicating the need for clinical assessment in the evaluation of clinical relevance (Table 3). In a different cohort of pneumonia patients, e.g., ICU patients, the concordance between OP- and LRT samples is likely to be different, due to a higher prevalence of *S. aureus* in this population (36).

Most divergent results between OP- and LRT samples were caused by detections of various Enterobacterales, which are of uncertain clinical importance in uncomplicated CAP (16, 37). There was a tendency that these bacteria occurred more frequently and with higher semi-quantitative values in the OP samples (Table 3), likely reflecting colonization of the URT. Furthermore, even though there was a small number of viral detections in this study, the data show a trend toward higher detection rates in LRT samples, resulting in a lower PPA. Indeed, and in line with previous investigations, four out of the 11 cases of rhino-/enterovirus were not detected in OP samples (12, 38). All of these patients reported respiratory tract symptoms of more than one week's duration, supporting observations that URT samples may be most sensitive in the acute phase of a viral RTI while LRT samples can be more sensitive at a later stage, especially with development of LRT infections (39, 40). A plausible bacterial pathogen was detected in all four patients, indicating that a secondary bacterial pneumonia caused their hospitalization. The utility of detecting a primary viral agent in such cases is uncertain.

The strengths of this study include a well-characterized study population with a CAP diagnosis based on predefined criteria, and the use of the FAP *plus* on paired OP- and LRT samples. A major quality is inclusion of patients in the ED where OP sampling preceded the LRT sample with a median time difference of only 65 (47–87) minutes, representing an equal basis for comparison with minimal impact of time and intravenous antibiotic use. Still, our study has some limitations. Our CAP population was limited to patients who can provide a high-quality LRT sample in the ED, which may have led to an exclusion of the most severe cases. Additionally, as typical CAP pathogens tend to colonize the URT more frequently in children compared to adults, our results may not be generalizable to children (41 - 43). Many of the bacterial targets included in the panel are not common causes of CAP. Still, we find it useful to show the agreement between OP- and LRT samples also for these targets, as one must relate to and evaluate their relevance when using the FAP *plus* in CAP patients. As part of the RCT study design, about half of the LRT samples and all the OP samples had been frozen upon testing by the FAP *plus*. However, we did not detect significant differences when we compared FAP *plus* results of fresh samples with re-analyzation of the same specimens after freezing (Table S2). The few discrepancies observed probably represented microbes that were present around the detection limit of the FAP *plus* assay. The FAP *plus* has been demonstrated to have a somewhat lower precision at some concentration values, and repeated measurements can thus likely produce slightly different results regardless of thawing (44). Several recent, large studies evaluating the FAP *plus* panel have used frozen samples (6, 9, 10, 45). Followingly, we find that the use of frozen LRT samples likely poses no or little disadvantage, with negligible impact on our results. Finally, it should be noted that the FAP *plus* is not regulatory approved for use in OP samples.

In conclusion, our study demonstrated that the FAP *plus* panel can detect the most common bacterial CAP pathogens from OP samples with high PPAs and excellent NPAs when compared with LRT samples. The PPV was excellent for *S. pneumoniae* and *H. influenzae*, but lower for bacteria that are less common causes of CAP (e.g., *S. aureus* and Enterobacterales), emphasizing the need for clinical evaluation of positive test results. Importantly, our study found higher PPAs for OP samples compared to previous studies that have used NP samples. Our findings suggest that OP samples analyzed on a syndromic PCR panel could represent an alternative approach for rapid microbiological testing in the ED, especially in patients where LRT samples are difficult to obtain.

## SUPPLEMENTAL MATERIAL

Supplemental tables jcm.00505-23-s0001.pdf Tables S1 and S2.

ASM does not own the copyrights to Supplemental Material that may be linked to, or accessed through, an article. The authors have granted ASM a non-exclusive, world-wide license to publish the Supplemental Material files. Please contact the corresponding author directly for reuse.
