# Supplementary material for: Diagnostic utility of oropharyngeal swabs as an alternative to lower respiratory tract samples for PCR-based syndromic testing in patients with community-acquired pneumonia
Source: J Clin Microbiol. 2023 Aug 16;61(9):e00505-23. doi: 10.1128/jcm.00505-23 (PMC10512787; doi:10.1128/jcm.00505-23)
Supplement: Supplemental tables — Tables S1 and S2. [file jcm.00505-23-s0001.pdf]

Supplementary material

Diagnostic utility of oropharyngeal swabs as an alternative to lower respiratory tract samples for PCR-based syndromic testing in patients with community acquired pneumonia

Running title: Oropharyngeal swabs for syndromic PCR-testing in CAP

Sondre Serigstad[a,b] #, Siri T. Knoop[c,d], Dagfinn L. Markussen[a,c], Elling Ulvestad[c,d], Rune O. Bjørneklett[a,b] Marit H. Ebbesen[d], Øyvind Kommedal[c,d] and Harleen M.S. Grewal[c,d]

Authors affiliations:

[a] Emergency Care Clinic, Haukeland University Hospital, NO-5021 Bergen, Norway

[b] Department of Clinical Medicine, University of Bergen, NO-5021 Bergen, Norway

[c] Department of Clinical Science, Bergen Integrated Diagnostic Stewardship cluster, University of Bergen, NO-5021 Bergen, Norway

[d] Department of Microbiology, Haukeland University Hospital, NO-5021 Bergen, Norway

# Corresponding author

# Corresponding author:

Sondre Serigstad, MD

Department of Clinical Medicine, University of Bergen, Bergen, Norway

Emergency Care Clinic, Haukeland University Hospital, NO-5021 Bergen, Norway

Email address: sondre.serigstad@helse-bergen.no

Phone number: +47 93862195

Table of contents (page number)

S1: Pairwise overview of detections by the Biofire FilmArray Pneumonia *plus* panel in the OP- and LRT samples ………. 3

S2: Results by the Biofire FilmArray Pneumonia *plus* panel in LRT samples before and after freezing………. 8

Supplementary material section one (S1)

Table S1. Pairwise overview of all detections by the Biofire FilmArray Pneumonia *plus* panel in the OP- and LRT samples. N=103.

Patient	LRT sample	OP sample
1	*H. influenzae*: ≥10^7	*H. influenzae*: ≥10^7
2	*E. coli*: ≥10^7<br>*E. cloacae*: 10^5	*E. coli*: ≥10^7<br>*A. baumaii:* 10^4
3	No detections	No detections
4	Rhino-/enterovirus<br>*S. pneumoniae*: ≥10^7<br>*H. influenzae*: 10^4<br>*S. aureus*: 10^4	Rhino-/enterovirus<br>*S. pneumoniae*: ≥10^7<br>*H. influenzae*: 10^5<br>*S. aureus*: 10^7
5	*H. influenzae*: ≥10^7<br>*S. pneumoniae*: 10^4<br>*S. aureus*: 10^6<br>*S. pyogenes*: ≥10^7	*H. influenzae*: ≥10^7<br>*S. pneumoniae*: 10^5<br>*S. aureus*: 10^5<br>*S. pyogenes*: ≥10^7
6	No detections	No detections
7	*H. influenzae*: 10^6<br>*S. pneumoniae*: 10^4	*H. influenzae*: 10^6<br>*S. pneumoniae*: 10^4
8	*H. influenzae*: 10^6	No detections
9	*M. catarrhalis:* 10^5	No detections
10	*S. pneumoniae*: 10^6<br>*S. aureus*: 10^4	*S. pneumoniae*: 10^5
11	*S. aureus:* 10^6	*S. aureus:* 10^6
12	No detections	No detections
13	*S. pneumoniae*: ≥10^7<br>*M. catarrhalis*: >10^7<br>*S. aureus*: 10^4<br>Rhino-/enterovirus	Rhino-/enterovirus
14	*Proteus spp.*: 10^4	*K. pneumoniae* group: ≥10^7<br>*Proteus spp*: ≥10^7<br>*S. aureus*: 10^6
15	*H. influenzae*: 10^4<br>*S. pneumoniae*: 10^5	*H. influenzae*: 10^4<br>*S. agalactiae*: 10^5
16	*H. influenzae*: ≥10^7<br>Adenovirus	*H. influenzae*: ≥10^7<br>Adenovirus
17	*M. catarrhalis*: >10^7<br>*K. oxytoca*: ≥10^7<br>*P. aeruginosa*: 10^5<br>*S. aureus*: ≥10^7	*M. catarrhalis*: >10^7<br>*K. oxytoca*: ≥10^7<br>*P. aeruginosa*: 10^4<br>*S. aureus*: ≥10^7
18	*P. aeruginosa:* ≥10^7	*P. aeruginosa:* ≥10^5
19	*E. coli*: 10^6	*E. coli*: ≥10^7<br>*S. marcescens*: 10^6
20	No detections	No detections

21	*S. agalactiae*: 10^5	*S. agalactiae*: 10^5
22	*H. influenzae*: 10^6	*H. influenzae*: 10^7
23	*S. aureus*: ≥10^7	*S. aureus*: ≥10^7
24	*S. marcescens*: 10^4	No detections
25	No detections	No detections
26	*P. aeruginosa*: ≥10^7	No detections
27	*S. pneumoniae*: ≥10^7<br>*S. aureus*: 10^5	*S. pneumoniae*: >10^6
28	No detections	*ACB-complex*: ≥10^7<br>*K. oxytoca:* ≥10^7<br>*S. aureus*: 10^5
29	*S. pneumoniae:* ≥10^7<br>Rhino-/enterovirus	*S. pneumoniae*: ≥10^5<br>Rhino-/enterovirus
30	*H. influenzae*: ≥10^7<br>*S. aureus*: 10^4	*H. influenzae*: ≥10^6<br>*S. aureus*: 10^6
31	No detections	*P. aeruginosa*: 10^6<br>*E. cloacae complex*: 10^4
32	*S. pneumoniae:* ≥10^7<br>*P. aeruginosa*: 10^6	*S. pneumoniae*: 10^4
33	*H. influenzae*: ≥10^7<br>Coronavirus (229E, OC43, HKU1, NL63)	*H. influenzae*: ≥10^4<br>Coronavirus (229E, OC43, HKU1, NL63)
34	*S. pneumoniae*: ≥10^7<br>*M. catarrhalis*: 10^4	*S. pneumoniae*: ≥10^7
35	*H. influenzae*: 10^6	*H. influenzae*: 10^5
36	*M. catarrhalis*: ≥10^7<br>*P. aeruginosa* : 10^5<br>Parainfluenza virus	*M. catarrhalis*: ≥10^7<br>*P. aeruginosa* : 10^5<br>Parainfluenza virus
37	*M. catarrhalis*: 10^4<br>*S. pneumoniae*: 10^6	*S. pneumoniae*: 10^5
38	*S. pneumoniae > 10^7*<br>*S. aureus:* 10^4<br>Rhino-/enterovirus	*S. pneumoniae*: 10^6<br>*S. marcescens*: 10^5<br>*S. aureus*: 10^5<br>Rhino-/enterovirus
39	*S. pneumoniae*: 10^5<br>Coronavirus (229E, OC43, HKU1, NL63)	Coronavirus (229E, OC43, HKU1, NL63)
40	*S. pneumoniae:* 10^6<br>*M. catarrhalis:* ≥ 10^7<br>RS-virus	*S. pneumoniae: 10^5*<br>*M. catarrhalis:* 10^4
41	*M. catharralis*: ≥10^7<br>*S. aureus*: 10^5<br>*S. agalactiae*: 10^5<br>Rhino-/enterovirus	*M. catarrhalis:* ≥ 10^7<br>*S. aureus:* ≥ 10^7<br>*S. agalactiae:* ≥ 10^7<br>*ACB-complex*: ≥10^7<br>Rhino-/enterovirus

42	*H. influenzae:* ≥ 10^7	*H. influenzae*: 10^6<br>*E. cloacae complex:* ≥ 10^7<br>*K. oxytoca:* ≥ 10^7
43	*H. influenzae*: ≥ 10^7<br>*S. aureus*: 10^5<br>RS-virus	*H. influenzae*: ≥ 10^7<br>*S. aureus*: 10^5<br>RS-virus
44	*E. coli* ≥ 10^7<br>*S. aureus* ≥ 10^7	*E. coli* ≥ 10^7<br>*S. aureus* ≥ 10^7
45	*S. pneumoniae:* ≥10^7<br>*H. influenzae*: 10^4<br>RS-virus	*S. pneumoniae:* ≥10^6<br>RS-virus
46	*H. influenzae* ≥ 10^7	*H. influenzae* ≥ 10^6
47	*S. pneumoniae*: ≥10^7<br>*H. influenzae*: 10^6<br>RS-virus	*S. pneumoniae*: ≥10^7<br>*H. influenzae*: 10^4<br>RS-virus
48	*H. influenzae* ≥ 10^7<br>Coronavirus (229E, OC43, HKU1, NL63)<br>Rhino-/enterovirus	*H. influenzae* ≥ 10^7
49	*S. pneumoniae*: ≥10^7<br>RS-virus	*S. pneumoniae*: ≥10^4<br>RS-virus
50	*H. influenzae:* ≥10^7<br>*E. coli*: 10^4<br>Rhino-/enterovirus	*H. influenzae:* ≥10^6<br>*E. coli*: 10^5
51	No detections	No detections
52	No detections	*S. aureus:* 10^4
53	No detections	*E. coli*: ≥ 10^7
54	*S. pneumoniae*: ≥10^7	*S. pneumoniae*: 10^6<br>*K. oxytoca*: 10^4
55	No detections	No detections
56	*E. coli:* ≥ 10^4<br>*S. pneumoniae*: ≥ 10^7<br>RS-virus	*E. coli:* ≥ 10^7<br>*S. pneumoniae*: ≥ 10^7<br>RS-virus<br>H. influenzae: 10^4
57	*H. influenzae*: ≥10^7	*H. influenzae >10^7*<br>*E. coli* ≥10^7
58	*P. aeruginosa*: ≥10^7<br>*S. aureus*: ≥10^7<br>*M. catarrhalis*: >10^7	*P. aeruginosa*: ≥10^7<br>*S. aureus*: ≥10^7<br>*M. catarrhalis*: 10^6
59	*S. pneumoniae*: ≥ 10^7	*S. pneumoniae*: 10^5
60	*M. catarrhalis*: ≥10^7<br>*S. pneumoniae*: 10^6	*M. catarrhalis*: 10^5<br>*S. pneumoniae*: 10^4<br>*E. coli*: > 10^7<br>*K. oxytoca*: > 10^7

61	*S. pneumoniae*: >10^7<br>*M. catarrhalis*: ≥10^7<br>*S. aureus*: 10^4	*S. pneumoniae*: ≥ 10^7<br>*M. catarrhalis*: 10^6<br>*S. aureus*: 10^4
62	*E. coli*: 10^4	*E. coli*: ≥ 10^7
63	*H. influenzae*: ≥ 10^7	*H. influenzae*: 10^4<br>*ACB-complex*: ≥10^7<br>*S. aureus*: 10^6
64	*H. influenzae*: ≥ 10^7<br>*M. catarrhalis*: ≥ 10^7	*H. influenzae*: ≥ 10^7<br>*M. catarrhalis*: 10^6
65	No detections	*K. pneumoniae group*: ≥ 10^7
66	*S. aureus*: 10^4	No detections
67	*H. influenzae*: ≥ 10^7<br>Rhino-/enterovirus	*H. influenzae*: 10^5
68	*H. influenzae*: 10^6	*H. influenzae*: ≥ 10^7
69	No detections	*K. oxytoca*: ≥ 10^7<br>*K. pneumoniae group*: ≥ 10^7
70	*H. influenzae*: 10^6<br>*M. catarrhalis*: ≥10^7<br>*S. aureus*: ≥10^7	*K. oxytoca*: ≥ 10^7<br>*M. catarrhalis*: ≥ 10^7<br>*S. aureus*: 10^6
71	*H.influenzae*: 10^5	*H. influenzae*: 10^6<br>*S. marcescens*: 10^5
72	*M. catarrhalis*: 10^4<br>Coronavirus (229E, OC43, HKU1, NL63)	*M. catarrhalis*: 10^5<br>Coronavirus (229E, OC43, HKU1, NL63)
73	*H. influenzae*: ≥ 10^7<br>*S. pneumoniae*: 10^6	*E. coli*: ≥ 10^7
74	Coronavirus (229E, OC43, HKU1, NL63)	Coronavirus (229E, OC43, HKU1, NL63)<br>*E. coli*: 10^6<br>*K. oxytoca*: 10^6
75	*H. influenzae*: ≥ 10^7	*H. influenzae*: 10^6
76	No detections	Coronavirus (229E, OC43, HKU1, NL63)
77	*H. influenzae*: 10^6<br>Rhino-/enterovirus	*H. influenzae*: 10^5
78	No detections	*S. marcescens*: 10^6<br>*S. aureus*: 10^6
79	No detections	*S. pneumoniae*: 10^5
80	*E. coli*: 10^6<br>*S. aureus*: 10^4<br>*K. pneumoniae group*: 10^4	*E. coli*: ≥ 10^7<br>*S. aureus*: ≥ 10^7
81	*M. catharrhalis*: ≥ 10^7	*M. catarrhalis: 10^4*<br>*S. aureus*: 10^5
82	*H. influenzae*: ≥ 10^7	*H. influenzae*: ≥ 10^7
83	*M. catarrhalis*: ≥ 10^7<br>Human metapneumovirus	*M. catarrhalis*: 10 ^4<br>Human metapneumovirus

84	No detections	No detections
85	*H. influenzae*: ≥ 10^7<br>*S. aureus*: 10^4<br>*M. catarrhalis*: 10^4<br>Influenza A virus	*H. influenzae*: ≥ 10^7<br>*S. aureus*: 10^6<br>*M. catarrhalis*: 10^4
86	*M. catarrhalis*: ≥ 10^7<br>*S. pneumoniae*: ≥ 10^7<br>*S. aureus*: 10^5	*M. catarrhalis*: ≥ 10^7<br>*S. pneumoniae*: ≥ 10^7<br>*S. aureus*: 10^5<br>*K. oxytoca*: 10^4
87	*H. influenzae*: ≥ 10^7	*H. influenzae*: ≥ 10^7
88	*H. influenzae*: ≥ 10^7	*K. oxytoca*: ≥10^7<br>*H. influenzae*: 10^6
89	*H. influenzae*: > 10^7<br>*K. pneumoniae group*: 10^4<br>*ACB-complex*: 10^5<br>*E. cloacae complex*: 10^4<br>Rhino-/enterovirus	*H. influenzae*: 10^6<br>*K. pneumoniae group*: 10^6<br>Rhino-/enterovirus
90	No detections	No detections
91	*H. influenzae*: 10^6	No detections
92	*H. influenzae*: ≥ 10^7	*H. influenzae: 10^5*<br>*S. marcescens*: ≥ 10^7
93	*H. influenzae*: ≥ 10^7	*H. influenzae*: 10^6
94	*H. influenzae*: ≥ 10^7<br>*S. pneumoniae*: ≥ 10^7	*H. influenzae*: 10^6<br>*S. pneumoniae*: 10^4
95	*H. influenzae*: ≥10^7	*H. influenzae*: 10^5<br>*S. agalactiae*: 10^5<br>*K. oxytoca*: 10^4
96	*H. influenzae*: ≥ 10^7	No detections
97	*H. influenzae*: ≥10^7	*H. influenzae*: 10^6
98	*S. aureus*: 10^5	No detections
99	*P. aeruginosa*: 10^6	*P. aeruginosa*: 10^4
100	No detections	No detections
101	*S. pneumoniae*: 10^6<br>*M. catarrhalis*: 10^4<br>Parainfluenza virus	*S. pneumoniae*: 10^4
102	*H. influenzae*: ≥10^7	*H. influenzae*: ≥10^7
103	*E. cloacae complex*: 10^4<br>*E. coli*: 10^6<br>Rhino-/enterovirus	*E. cloacae complex*: ≥ 10^7<br>*E. coli*: ≥ 10^7<br>*S. marcescens*: ≥10^7<br>Rhino-/enterovirus

Abbreviations: OP, oropharyngeal; LRT, lower respiratory tract.

Supplementary material section two (S2)

Table S2. Results by the Biofire FilmArray Pneumonia *plus* panel in lower respiratory tract samples before and after freezing at (-80°C). Pairwise presentation. Discordance in detections are marked in **bold**.

Fresh sample (analyzed before freezing)	Analyzed after freezing (-80°C)
H. influenzae≥ 10^7<br>**Coronavirus (229E, OC43, HKU1, NL63)**<br>**Rhino-/enterovirus**	*H. influenzae* ≥ 10^7
H. influenzae ≥ 10^7<br>*E. coli* 10^4<br>Rhino-/enterovirus	*H. influenzae* ≥ 10^7<br>*E. coli* 10^4<br>Rhino-/enterovirus
No detection	***S. aureus* 10^4**
No detection	No detection
S. pneumoniae ≥ 10^7	*S. pneumoniae* ≥ 10^7
M. catarrhalis ≥ 10^7<br>*S. marcescens* 10^4<br>*S. pneumoniae* ≥ 10^7	*M. catarrhalis* ≥ 10^7<br>*S. marcescens* 10^5<br>*S. pneumoniae* ≥ 10^7
No detection	No detection
No detection	No detection
H. influenzae 10^6<br>*M. catarrhalis* ≥ 10^7<br>*S. aureus* ≥ 10^7	*H. influenzae* 10^5<br>*M. catarrhalis* ≥ 10^7<br>*S. aureus* 10^6
H. influenzae 10^5	*H. influenzae* ≥ 10^7
M. catarrhalis 10^4<br>Coronavirus (229E, OC43, HKU1, NL63)	*M. catarrhalis* 10^4<br>Coronavirus (229E, OC43, HKU1, NL63)
No detection	**Coronavirus (229E, OC43, HKU1, NL63)**
H. influenzae 10^6<br>Rhino-/enterovirus	*H. influenzae* ≥ 10^7<br>Rhino-/enterovirus
No detection	No detection
E. coli 10^6<br>*K. pneumoniae* 10^4<br>*S. aureus* 10^4	*E. coli* ≥ 10^7<br>*K. pneumoniae* 10^4<br>*S. aureus* 10^6
H. influenzae ≥ 10^7	*H. influenzae* ≥ 10^7
No detection	No detection
H. influenzae ≥ 10^7<br>*ACB-complex* 10^5<br>*K. pneumoniae* 10^4<br>***Enterobacter cloacae-complex* 10^4**<br>Rhino-/enterovirus	*H. influenzae* ≥ 10^7<br>*ACB-complex* 10^5<br>*K. pneumoniae* 10^4<br>Rhino-/enterovirus
No detection	No detection
H. influenzae 10^6	*H. influenzae* 10^6
H. influenzae ≥ 10^7<br>*S. pneumoniae* ≥ 10^7	*H. influenzae* ≥ 10^7<br>*S. pneumoniae* ≥ 10^7
H. influenzae ≥ 10^7	*H. influenzae* > 10^7<br>***S. agalactiae* 10^4**
